# Polyphenols (S3) Isolated from Cone Scales of *Pinus koraiensis* Alleviate Decreased Bone Formation in Rat under Simulated Microgravity

**DOI:** 10.1038/s41598-018-30992-8

**Published:** 2018-08-24

**Authors:** Yan Diao, Bin Chen, Lijun Wei, Zhenyu Wang

**Affiliations:** 1 Harbin Institute of Technology, Harbin, China; 2 Key Laboratory of Space Nutrition and Food Engineering, China Astronauts Research and Training Center, Beijing, China; 3 State Key Laboratory of Space Medicine Fundamentals and Application, Chinese Astronaut Research and Training Center, Beijing, China

## Abstract

In order to screen out an effective bone loss protectant from natural plant polyphenol and to elucidate the mechanism of the plant polyphenols that alleviate bone loss under simulated microgravity, the proliferation activities of 9 total polyphenol extracts from natural product (TPENP) on osteoblasts were measured. Polyphenols (S3) was isolated from total polyphenols of cone scales from *pinus koraiensis* (Korean pine). ALP activity in osteoblasts and MDA level in femur were measured. Mechanical properties and microstructure of the distal cancellous region of the femur in rat were tested. Various bone metabolism markers, enzymes activity and genes expression were also analyzed. The results showed that S3 has the highest activity of osteoblast proliferation. S3 promoted ALP activity in osteoblasts, enhanced mechanical properties and microstructure of the distal cancellous region of femur in rat, decreased MDA level, elevated the serum concentration of BALP, PINP and activities of SOD, CAT, GSH-Px in femur under simulated microgravity. In addition, S3 enhanced the expression of NRF-2, β-catenin, p-GSK3-β, OSX, RUNX2, Osteonectin, Osteocalcin, ALP and collagen I. These results indicated that S3 can alleviated bone loss induced by simulated microgravity through abate the inhibition of the oxidative stress on Wnt/β-catenin signaling pathway.

## Introduction

Bone loss which is characterized by unstable bone structure and reduced bone mass can be induced by long term spaceflight^1^. Studies of the bone density of astronauts found that after long duration spaceflight, the cancellous bone formation of astronauts was reduced, bone metabolism was abnormal, and there were various degrees of bone mineral density and bone mineral loss^2,3^. By marking the rat bone with tetracycline, the cortex and cancellous bone formation were found decreased after spaceflight^4–6^. Unlike bone loss on ground, the occurrence and development of weightlessness bone loss has certain site selectivity, and the bone loss is difficult to recover after return to the ground^7^. Therefore, space medicine has been devoted to the study of effective protection against bone loss under microgravity. Based on the current theory, there are two ways to deal with bone loss in microgravity environment, one is to carry out resistance training, and the other is to do the drug intervention. But confrontational training equipment for alleviating bone loss caused by microgravity is weak, and chemical drug have serious side effects. Therefore, the search for effective bone loss protectants is one of the most important methods for human body to adapt to microgravity environment.

Plants are the main source of drugs for the treatment of various diseases, so more and more attention has been paid to the search for new powerful bone loss protectants from plants. Polyphenols are a group of compounds with similar chemical structure, and are structurally characterized by one and more than one phenolic hydroxyl unit^8^. Polyphenols from natural plants have the characteristics of small dose and strong effect, diverse biological activity and etcetera. According to the characteristics of polyphenolic compounds, the plant polyphenols can be divided into phenolic acids, flavonoids, stilbenes and wood flour compounds^9,10^. Current studies have shown that natural polyphenols have many physiological functions, including improved cardiovascular and cerebrovascular function^11^, effects on nervous system^12,13^, anti-inflammatory effects^14^, significant anticancer^15,16^ and anti-aging^17^, etc. The daily intake of a certain amount of natural polyphenols can effectively prevent and inhibit the occurrence of disease^18,19^. Therefore, the polyphenols from natural plants has the potential to develop protectant for bone loss caused by a microgravity environment.

Studies have found that Pycnogenol® (extracts of *Pinus pinaster Ait*. bark) has the effect of anti-bone loss^20^, and its main component is plant polyphenols. *Pinus koraiensis* (Korean pine) and *Pinus pinaster Ait*. are both belongs to pinus lambertiana, so polyphenols in Korean pine have the potential to become a bone loss protectant. In *The Pharmacopoeia of the People’s Republic of China*, most of the medicinal parts of the Korean pine were root, stem and bark, however, without records of the cone scale. Cone scales is considered a type of trash in the traditional concept and can be buried or burned. But, the degradation rate of landfill is slowly, while incineration will cause serious environmental pollution. Therefore, there is no appropriate method for handling the scales of Korean pine cone that had been considered as “junk” until now. If weightlessness bone loss protectant can be developed from cone scale of Korean pine, the problem of environmental pollution can be solved as killing two birds with one stone. Searching weightlessness bone loss protectant from polyphenols in natural plants is a new breakthrough relative to the current development of bone loss protectants. In this paper, study will be started with the obtaining protectant to alleviate bone loss under microgravity from polyphenols in scales of Korean pine cone.

Firstly, the research value of total polyphenol extract in scale of Korean pine cone will be determined through comparison of cone scale from Korean pine and herbs that were commonly used in clinic and medicinal & edible in *The Pharmacopoeia of the People’s Republic of China*, while the contribution to osteoblasts proliferation activity of the total polyphenol extracts shall be screened and studied, and the polyphenol section with the highest activity of osteoblasts proliferation should be searched through isolation and purification. Secondly, whether polyphenol section from cone scale of Korean pine can alleviate the decreased ALP activity in osteoblasts should be verified under simulated microgravity *in vitro* studies, so that whether the Korean pine polyphenols section had the potential capacity to alleviate bone loss caused by the microgravity can be preliminarily verified. Thirdly, *in vivo* study, whether the polyphenols section can alleviated bone loss induced by microgravity should be tested through the analysis of mechanical properties of femur and μ-CT scanning of cancellous of distal femur in rats, in order to obtain natural plant extracts that had the potential capacity to alleviate bone loss under microgravity. Finally, the mechanism that polyphenol section from Korean pine alleviated bone loss induced by simulated microgravity should be elucidated through biochemical indicators in serum and femur and the signaling pathways.

## Results

### Comparison of proliferation activities of 9 TPENP on osteoblasts

**Figure 1:**
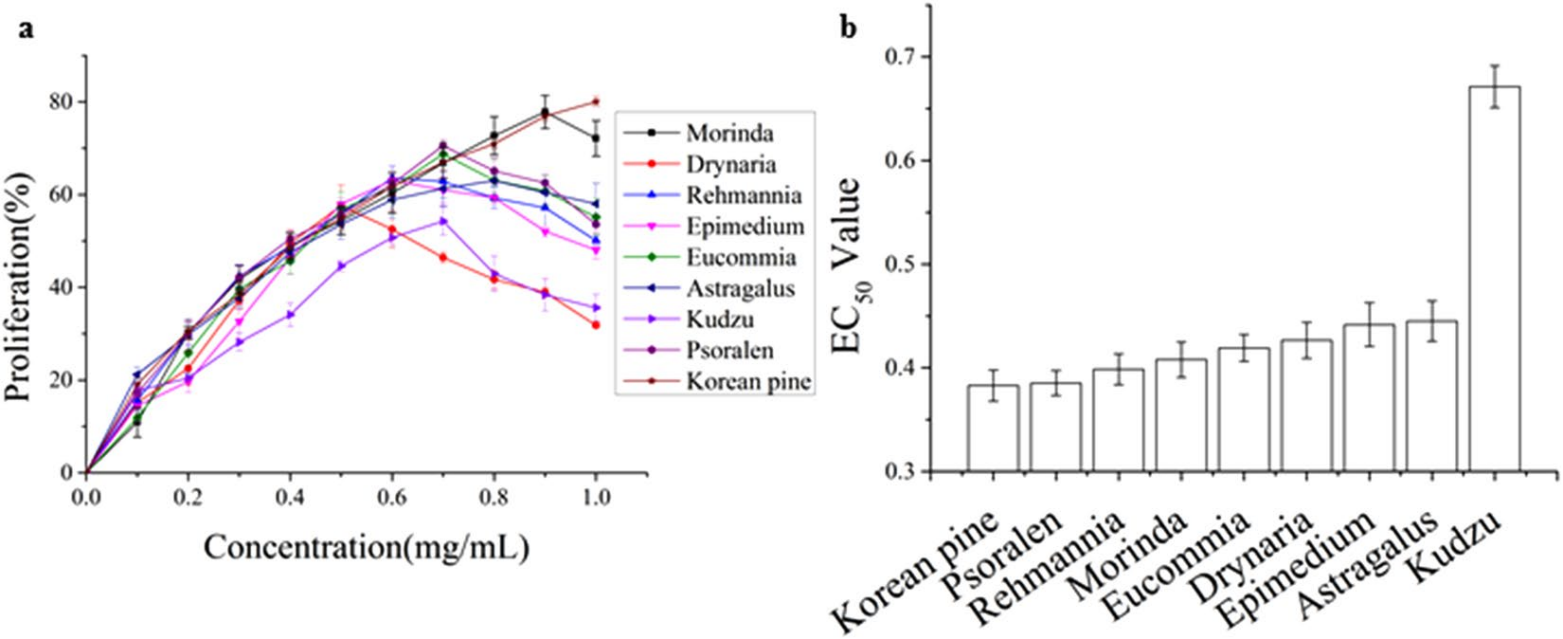
Comparison of proliferation activities of 9 TPENP on osteoblasts. (**a**) Proliferation curve of TPENP on osteoblasts, cell proliferation was evaluated by MTT assay. (**b**) EC_50_ value of proliferation of 9 TPENP on osteoblasts. The statistical results shown represent the means ± SD; (n = 3).

The number of osteoblast played a direct role in bone forming so the screening of proliferation activities of 9 total polyphenol extracts from natural product (TPENP) on osteoblasts was carried out first. Varying degrees of proliferation activity on osteoblasts were identified for 9 TPENP and The EC_50_ values of proliferation activities of 9 TPENP on osteoblasts were showed in Fig. 1b. EC_50_ values were arranged in ascending order: Korean pine, Psoralen, Rehmannia, Morinda, Eucommia, Drynaria, Epimedium, Astragalus and Kudzu. The total polyphenols of Korean pine had the highest proliferation activity on osteoblasts through comparison of EC_50_ values. So the Korean pine was selected for research in this study.

### Isolation and purification of total polyphenols of Korean pine and comparison of proliferation activities of S3, S4 on osteoblasts

**Figure 2:**
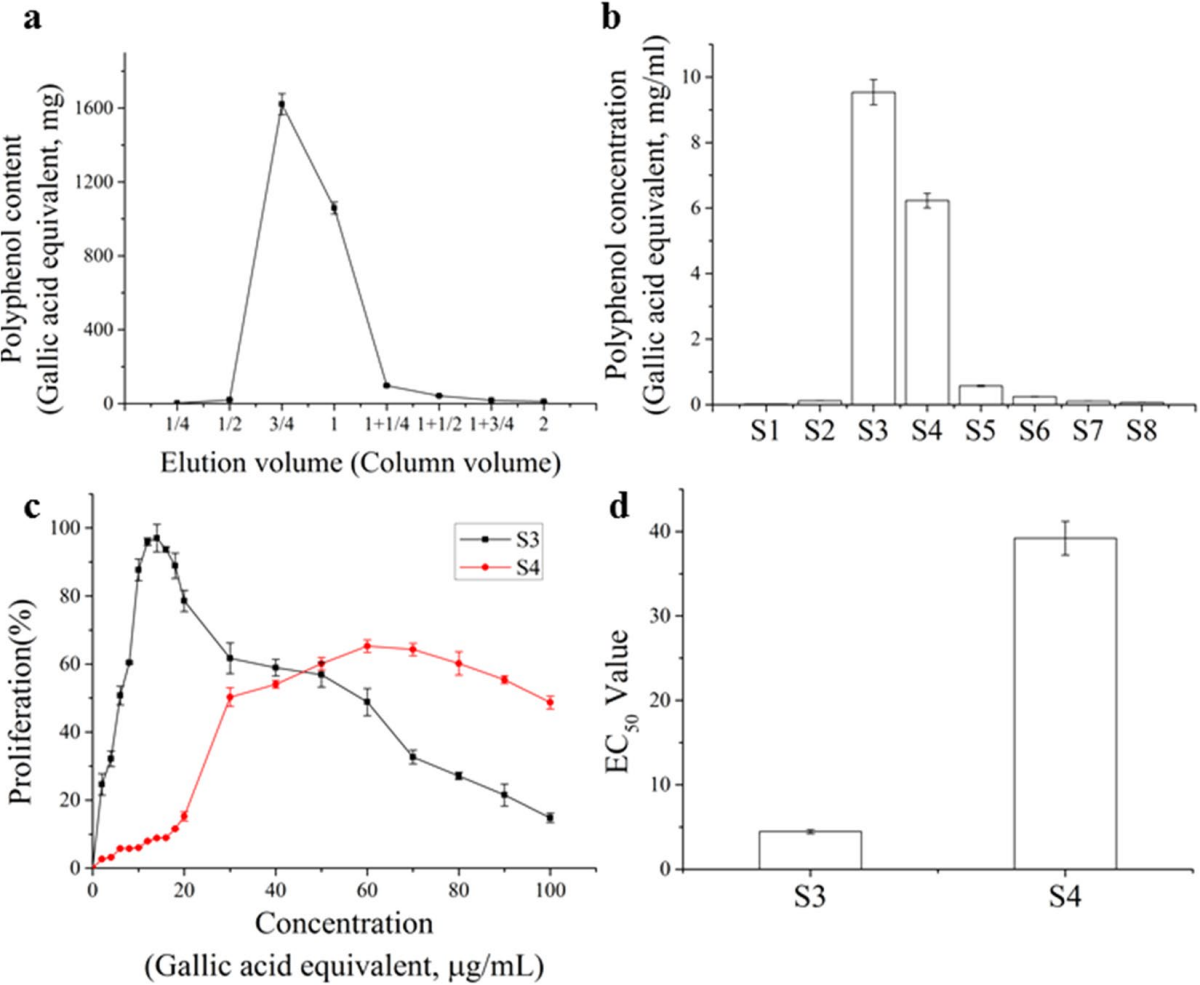
Isolation and purification of total polyphenols in cone scale of Korean pine and comparison of proliferation activities of S3, S4 on osteoblasts. (**a**) Elution curves of column chromatography of total polyphenols in Korean pine. (**b**) Polyphenol content in S1-S8 after isolation and purification of total polyphenols in cone scale of Korean pine. (**c**) The proliferation curve of S3, S4 on osteoblasts, cell proliferation was evaluated by a MTT assay. (**d**) The EC50 value of proliferation of S3, S4 on osteoblasts. The statistical results shown represent the means ± SD; (n = 3).

In order to screen out compounds with similar chemical structure that promote osteoblasts proliferation, total polyphenols of Korean pine were isolated and purificated for this study. With the increasing volume of elution, the content of polyphenols in the eluent increased first at 1/2 Column volume and then decreased at 3/4 Column volume (Fig. 2a). In column chromatography elution, each 1/4 column volume of eluent was counted as one section, so the total polyphenol of Korean pine was divided into eight sections of S1–S8, and the content of polyphenols in S3 was the highest, (9.54 ± 0.39 mg/ml), and S4 followed (6.23 ± 0.22 mg/ml), while polyphenols in the remaining section were less than 0.6 mg/ml (Fig. 2b). This data indicated that the polyphenols isolated from Korean pine that promote osteoblasts proliferation were mainly in S3 and S4, while the main components in other sections were not polyphenols. Therefore, the abilities of S3 and S4 to promote the proliferation of osteoblasts were compared. The concentration of S3 and S4 of highest relative proliferation rate of osteoblasts was 16 μg/ml and 60 μg/ml, respectively (Fig. 2c). The comparison of EC_50_ value showed that the proliferation activity of S3 on osteoblasts was better than S4 (Fig. 2d), so S3 was selected for further research.

### Decreased ALP activity in osteoblasts was alleviated by S3 under simulated microgravity

**Figure 3:**
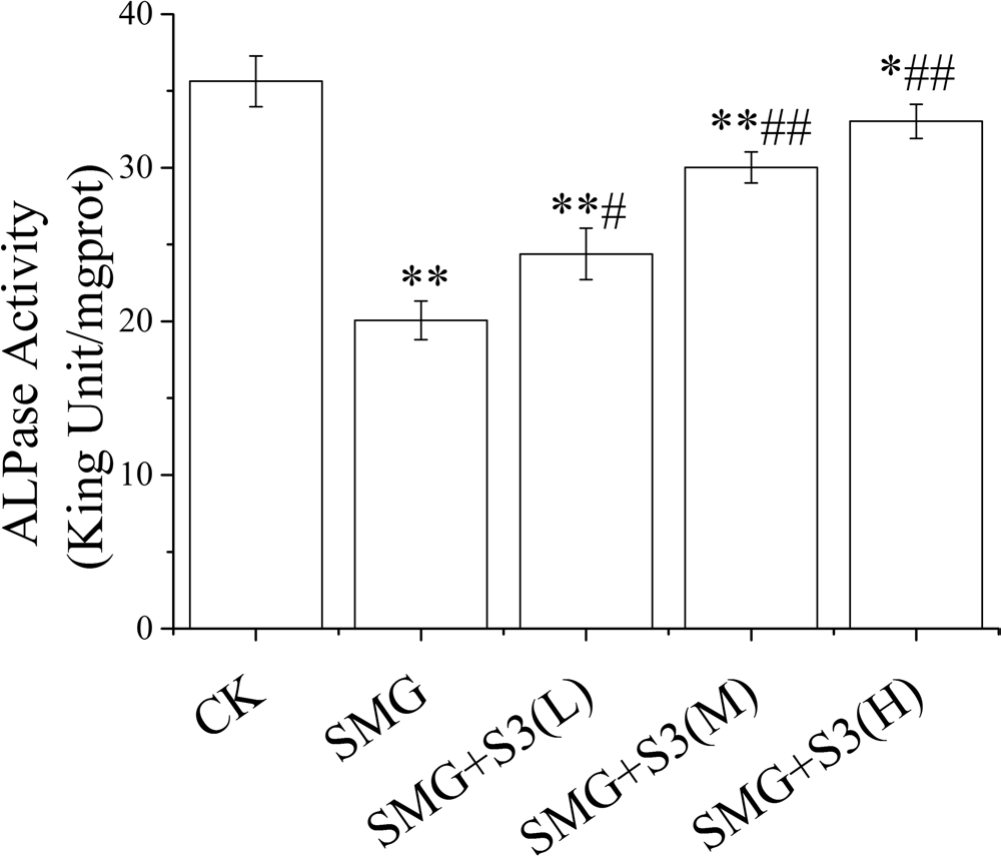
Decreased ALP activity was alleviated by S3 in osteoblasts under simulated microgravity. Rotating Wall Vessel (2D-RWVS, China Astronauts Research and Training Center) was used to set up a simulated microgravity model *in vitro*. CK: Ground control, SMG: simulated microgravity, S3(L): low dose of S3, S3(M): middle dose of S3, S3(M): high dose of S3. The statistical results shown represent the means ± SD; (n = 3). vs. the SMG group ^#^*P* < 0.05, ^##^*P* < 0.01; vs. the CK group *P < 0.05, **P < 0.01.

In order to verify whether S3 was able to improve the activity of osteoblasts under microgravity by Rotating Wall Vessel (2D-RWS), effect of S3 on ALP activity was studied (Fig. 3). Under simulated microgravity for 72 h, ALP activity in osteoblasts was significantly decreased. S3 could elevate ALP activity at a way of dose-effect, however, high-doses S3 still failed to achieve ALP activity to normal levels in osteoblast under simulated microgravity. This result indicated that the decreased osteoblast activity which induced by simulated microgravity could be alleviated by S3.

### The decreased mechanical properties of the femur in rats were alleviated by S3 under hind-limb unloading (HLS)

**Figure 4:**
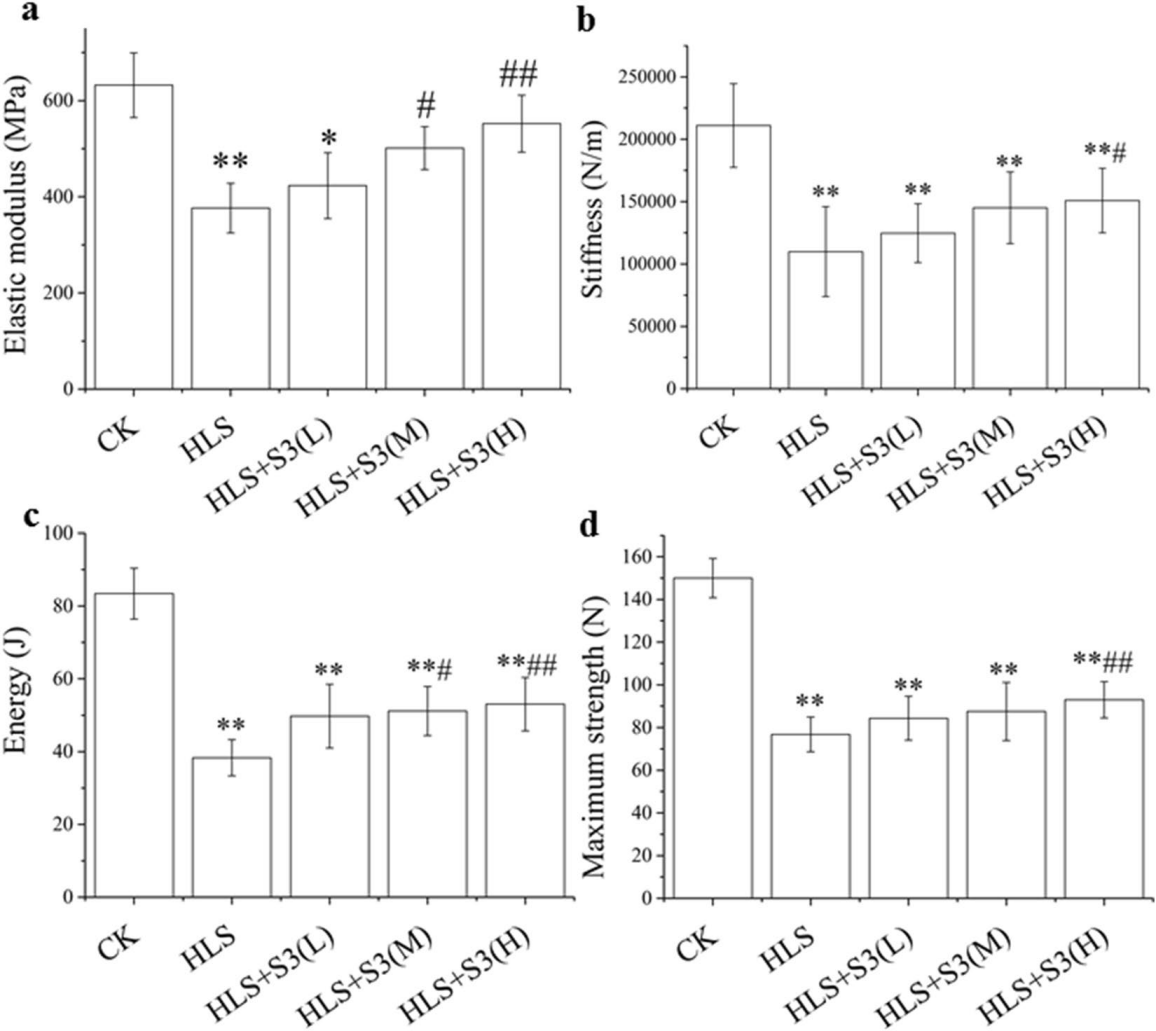
Decreased mechanical properties of the femur were alleviated by S3 in rats under HLS. Hind limbs unloading (HLS) method was used to set up a simulated microgravity model *in vivo*. CK: Ground control; HLS: Rats were suspended, unloaded with −30° downward head tilting; S3(L): low dose of S3, S3(M): middle dose of S3, S3(M): high dose of S3. (**a**) Elastic modulus (MPa) was determined by 3-point bending test. (**b**) Stiffness (N/m) was determined by 3-point bending test. (**c**) Energy (J) was determined by 3-point bending test. (**d**) Maximum strength (N) was determined by 3-point bending test. The statistical results shown represent the means ± SD; (n = 7). vs. the HLS group ^#^*P* < 0.05, ^##^*P* < 0.01; vs. the CK group *P < 0.05, **P < 0.01.

To test whether bone loss induced by hind limb unloading model (HLS) was alleviated by S3 in rats. Mechanical properties of the femur in rats were investigated. 40 Femurs were harvested from 5 group (CK, HLS, HLS + S3(L), HLS + S3(M), HLS + S3(H)) SD rat. Compared with CK, the elastic modulus, stiffness, energy and maximum strength of femur were significantly decreased after HLS in rats (Fig. 4). The decreased mechanical properties of femur were significantly alleviated by middle and high doses of S3 in different levels, and the high-dose S3 had the best effect. The results showed that the decreased mechanical properties of femur could be alleviated by high-dose S3 in rat after HLS.

### Change of the cancellous in distal of femur in rats was alleviated by S3 under HLS

**Figure 5:**
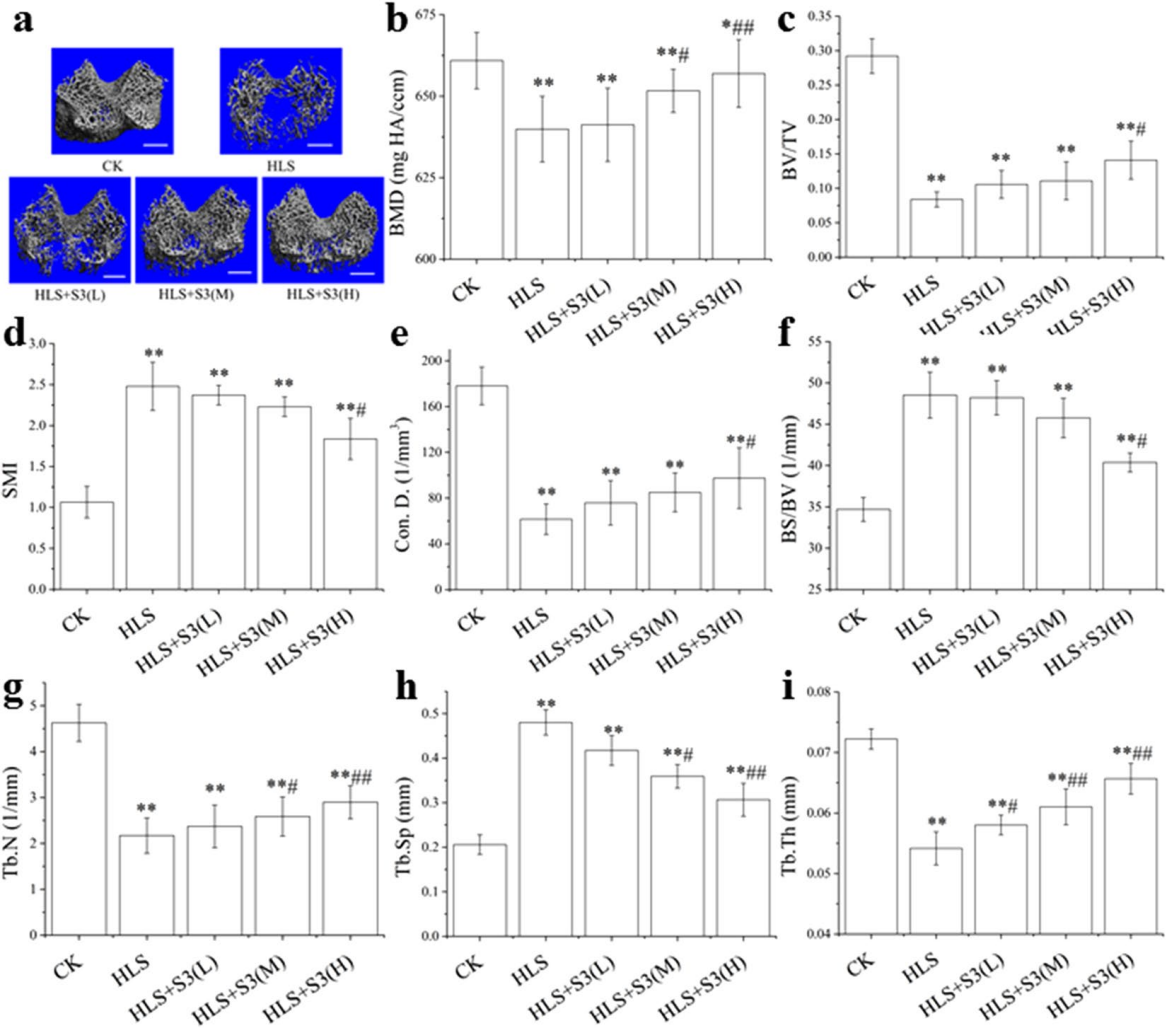
Change of the cancellous in distal of femur was alleviated by S3 in rats under HLS. cancellous: one 2.1-mm-thick trabecular bone chip under the epiphyseal plate in the lower end of the femur was selected as the region of interest (ROI), scale: 1 mm. CK: Ground control; HLS: Rats were suspended, unloaded with −30° downward head tilting; S3(L): low dose of S3, S3(M): middle dose of S3, S3(M): high dose of S3. (**a**) 3D reconstruction images of cancellous of ROI in femur. (**b**) BMD, bone mineral density (mg HA/ccm), refers to the total BMD of the ROI was determined by micro-CT. (**c**) BV/TV, bone volume fraction, refers to the ratio of bone volume to tissue volume was determined by micro-CT. (**d**) SMI, structure model index, is a method for determining the plate- or rod-like geometry of trabecular structures was determined by micro-CT. (**e**) Con.D, connectivity density shows the number of connections in the trabecular networks, was determined by micro-CT. (**f**) BS/BV, bone surface to bone volume (1/mm), refers the content of bone tissue in the sample Reflect the content of bone tissue in the sample. (**g**) Tb.N, trabecular number (1/mm) was determined by micro-CT. (**h**) Tb.Sp, trabecular separation (mm) was determined by micro-CT. (**i**) Tb.Th, trabecular thickness (mm) was determined by micro-CT. The statistical results shown represent the means ± SD; (n = 7). vs. the HLS group ^#^*P* < 0.05, ^##^*P* < 0.01; vs. the CK group *P < 0.05, **P < 0.01.

In order to study the effect of S3 on the microstructure in femur of rats after HLS, 3D images of the 2 mm region above the growth plate of the distal femur was reconstructed and analyzed (Fig. 5a). The contours of cancellous area from distal femur (CADF) almost disappeared and the order of tissue was significantly reduced after HLS. With the increase of S3 dosage, the contours of CADF became clear, and the order of tissue gradually increased, and the high-dose S3 had the best alleviating effect after HLS.

BMD, BV/TV, Con.D., Tb.N., Tb.Th. of CADF in rat were significantly decreased compared with CK (Fig. 5b,c,e,g,i) while SMI, BS/BV, Tb.Sp. were significantly increased (Fig. 5 d,f,h) through quantitative analysis of region of interest ROI after HLS. Low-dose S3 had no significant effect on CADF in rats after HLS except Tb.Th. Middle-dose S3 could significantly increase BMD and Tb.N. and decreased Tb.Sp. The reduction of BMD, BV/TV, Con.D., Tb.N., Tb.Th., and the rise of BS/BV, SMI, Tb.Sp. of CADF induced by HLS could be significantly alleviated by high-dose S3. These results suggest that the impacts of CAFD after HLS could be alleviated by S3 in rat, and the high dose S3 had the best effect among the three dose groups.

### Bone loss was alleviated by S3 through enhance bone formation capability under HLS

**Figure 6:**
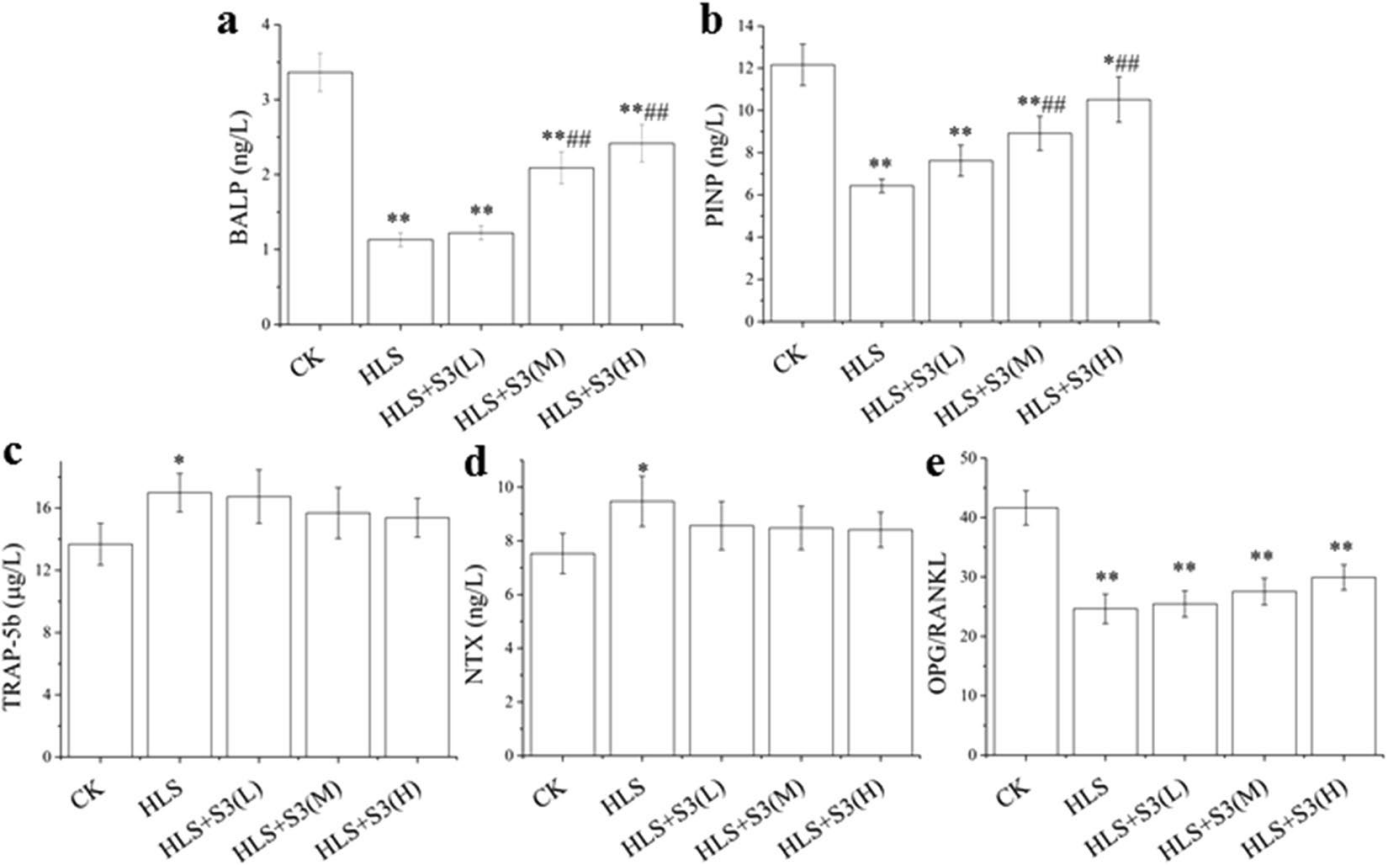
Effects of S3 on serum concentration of BALP, PIPN, TRAP-5b, NTX and OPG/RANKL in femur of rats under HLS. CK: Ground control; HLS: Rats were suspended, unloaded with −30° downward head tilting; S3(L): low dose of S3, S3(M): middle dose of S3, S3(M): high dose of S3. Serum concentrations of (**a**) BALP (μg/L), (**b**) PIPN (μg/L), (**c**) TRAP-5b (μg/L), (**d**) NTX (μg/L) were tested. (**e**) The value of OPG/RANKL in femur of rats. The statistical results shown represent the means ± SD; (n = 7). vs. the HLS group ^#^*P* < 0.05, ^##^*P* < 0.01; vs. the CK group *P < 0.05, **P < 0.01.

To investigate why the femoral bone loss could be alleviated by S3 in rats after HLS, the level of BALP, PIPN, TRAP-5b and NTX in serum were tested (Fig. 6). After HLS, BALP and PINP in serum of rat were significantly decreased (Fig. 6a,b). BALP and PINP were significantly increased after rats were gavage with middle-dose and high-dose S3 under HLS, and high-dose S3 was the best. However, the results of TRAP-5b and NTX in rat serum were very different, no significant effect of S3 on TRAP-5b and NTX in serum of rats after HLS (Fig. 6c,d). To further investigate whether S3 could alleviate bone loss by the inhibition of osteoclast activity through OPG/RANKL/RANK signaling pathway after HLS, OPG and RANKL in femur of rat were tested (refer to Fig. 6e). The OPG/RANKL value decreased significantly in femur of rat after HLS, and there were no significant change of all three dose of S3 on the OPG/RANKL value. These results suggest that S3 alleviate the bone loss in femur might through enhancing bone formation capability in rat after HLS.

### Increased oxidative stress of femur in rats was alleviated by S3 under HLS

**Figure 7:**
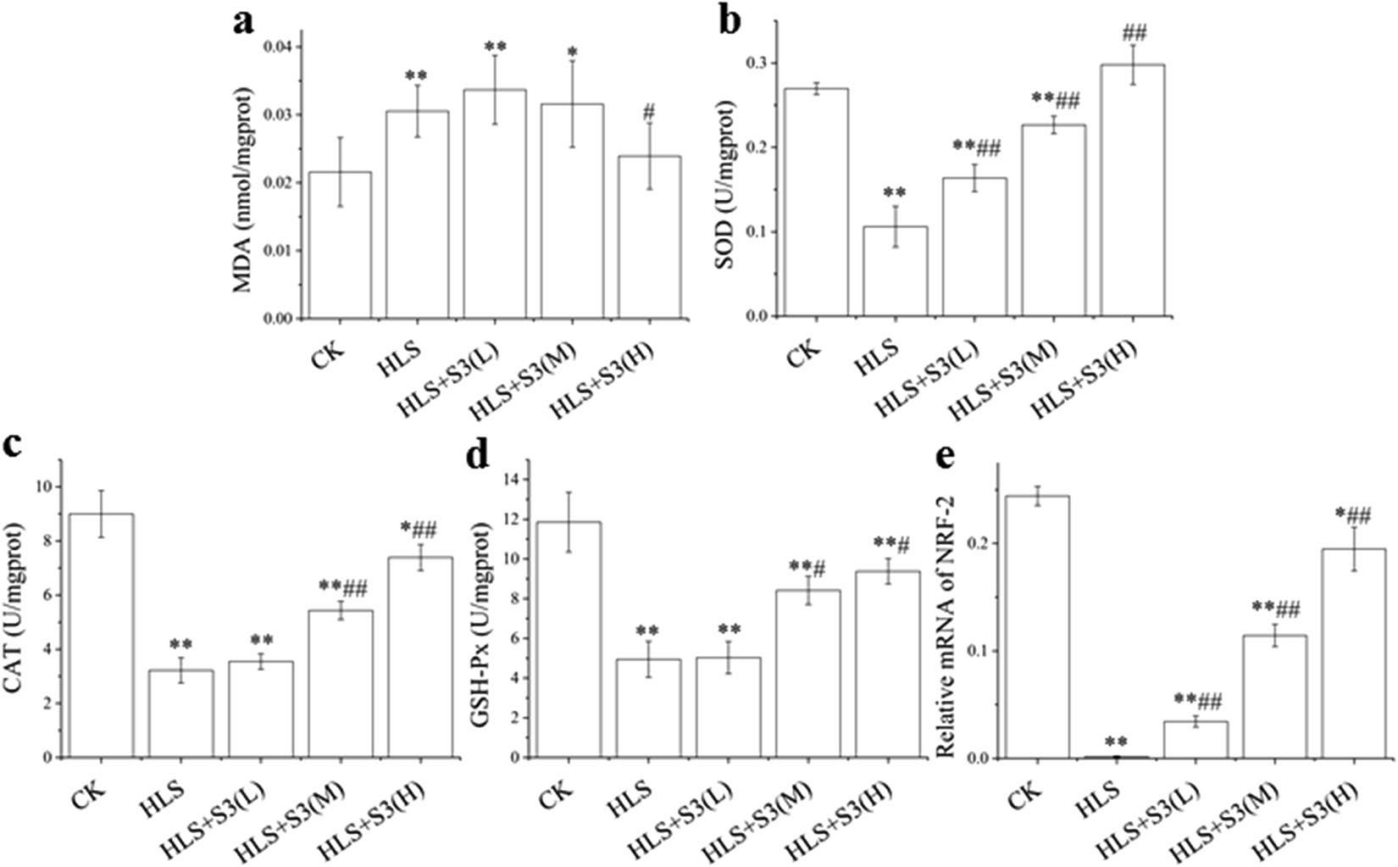
The increased MDA and the decreased SOD, CAT, GSH-Px and NRF-2 in femur of rat were alleviated by S3 under HLS. CK: Ground control; HLS: Rats were suspended, unloaded with −30° downward head tilting; S3(L): low dose of S3, S3(M): middle dose of S3, S3(M): high dose of S3. (**a**) The level of MDA (nmol/mgport). (**b**) The activity of SOD (U/mgport). (**c**) The activity of CAT (U/mgport). (**d**) The activity of GSH-Px (U/mgport). (**e**) qRT-PCR analysis of the changes in the mRNA expression of NRF-2 in rat femoral tissue. The statistical results shown represent the means ± SD; (n = 7). vs. the HLS group ^#^*P* < 0.05, ^##^*P* < 0.01; vs. the CK group *P < 0.05, **P < 0.01.

Bone loss is often associated with oxidative stress. Therefore, in order to study why bone loss could be alleviated by S3 after HLS, MDA, SOD, CAT, GSH-Px and NRF-2 in femur were tested (Fig. 7). After HLS, MDA in femur was significantly increased while the activity of SOD, CAT and GSH-Px were decreased significantly, as well as the expression of NRF-2. The increased MDA and decreased activities of SOD, CAT, GSH-Px could be significantly reduced and promoted by High-dose S3, respectively (Fig. 7a–d). The expression of NRF-2 was increased significantly by three different doses of S3 in dose-effect relationship (Fig. 7e). These results indicate that oxidative stress occurs in femoral tissue after rat suffered HLS, and this oxidative stress could be alleviated by S3.

### The decreased β-catenin, p-GSK3-β, RUNX2, OSX, Osteonectin, Osteocalcin, Collagen I and ALP expression in femur was alleviated by S3 under HLS

**Figure 8:**
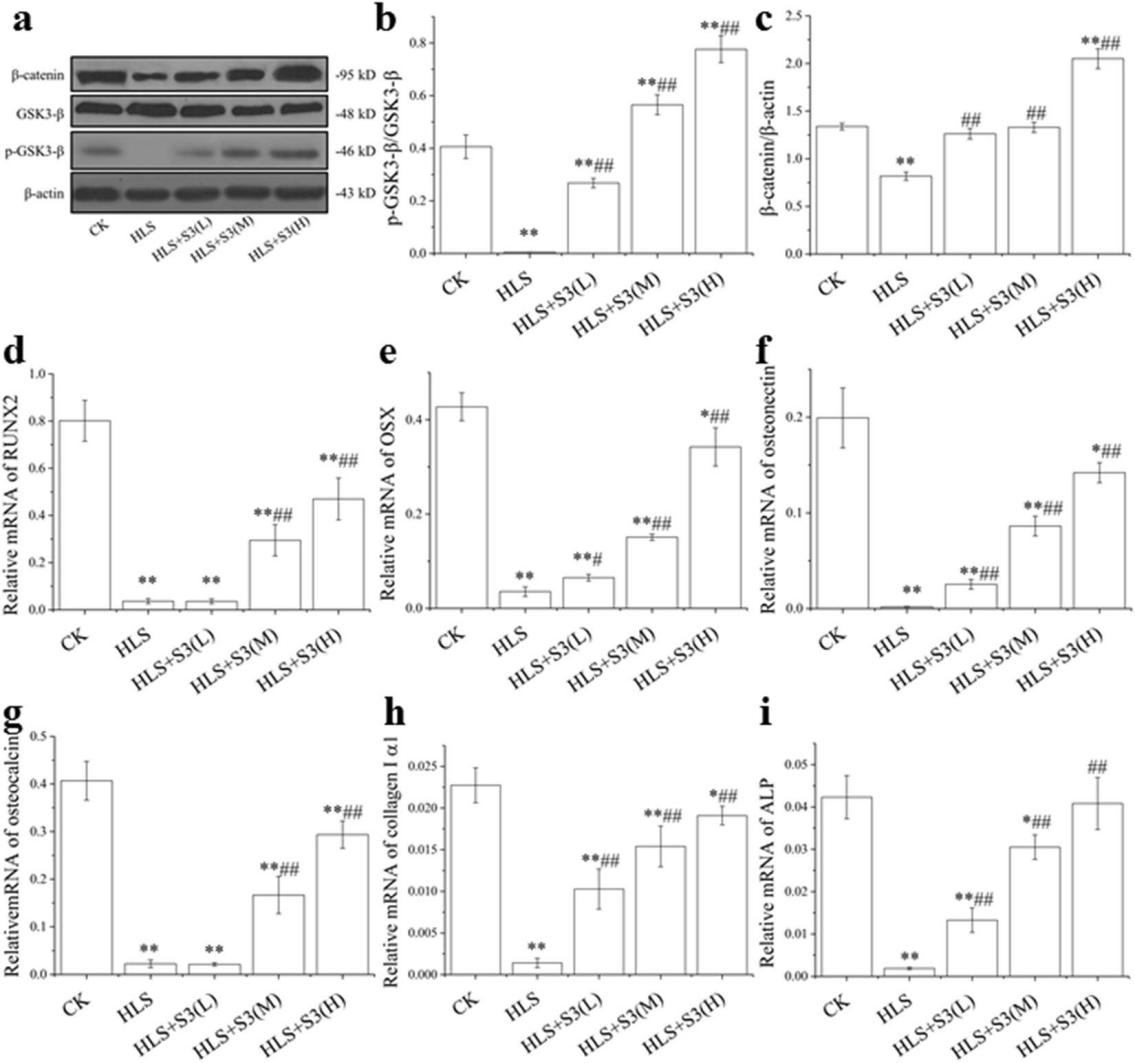
Decreased expression of β-catenin, GSK3-β, p-GSK3-β, RUNX2, OSX, Osteonectin, Osteocalcin, Collagen I α1 and ALP in femur of rats were alleviated by S3 under HLS. CK: Ground control; HLS: Rats were suspended, unloaded with −30° downward head tilting; S3(L): low dose of S3, S3(M): middle dose of S3, S3(M): high dose of S3. (**a**–**c**) Western blot analysis of the changes in β-catenin, GSK3-β and p-GSK3-β protein levels in rat femoral tissue. The qRT-PCR analysis of the changes in the mRNA expression in rat femoral tissue, including (**d**) RUNX2, (**e**) OSX, (**f**) Osteonectin, (**g**) Osteocalcin, (**h**) Collagen I α1, (**i**) ALP. The Western blot gels were cropped from different gels (β-catenin, GSK3-β, p-GSK3-β and β-actin) and the full-length gels are presented in Supplementary Fig. S1. The samples derive from the same experiment and that blots were processed in parallel. The statistical results shown represent the means ± SD; vs. the HLS group ^#^*P* < 0.05, ^##^*P* < 0.01; vs. the CK group *P < 0.05, **P < 0.01.

Wnt-β-catenin signaling pathway which plays an important role in bone formation can be inhibited by oxidative stress. In order to further study the mechanism of S3 in alleviating bone loss, the expression of β-catenin, GSK3-β, p-GSK3-β, RUNX2, OSX, Osteonectin, Osteocalcin, Collagen Iα1 and ALP were tested (Fig. 8). After HLS, β-catenin p-GSK3-β/GSK3-β decreased significantly, and with the S3 dose increased, p-GSK3-β and the β-catenin increased (Fig. 8a–c). The expression of RUNX2, OSX, Osteonectin, Osteocalcin, Collagen Iα1 and ALP in femur were significantly decreased in femur of rats after HLS, and were significantly increased by both middle-dose and high-dose S3 (Fig. 8d–i). These results suggest that the inhibition of Wnt-β-catenin signaling pathway and the decreased osteogenic genes include RUNX2, OSX, Osteonectin, Osteocalcin, Collagen Iα1 and ALP, in femur of rats induced by HLS could be alleviated by S3.

## Discussion

Bone loss is the result of disruption in homeostasis between osteogenesis of osteoblasts and bone resorption of osteoclasts^21,22^. The mostly current clinical drugs treat bone loss through inhibiting the differentiation or activity of osteoclasts which make bone homeostasis to the direction of bone formation. However, the medicines developed in this way have serious side effects^23^. Osteoblasts have proliferative ability, while osteoclasts are terminally differentiated cells and lose proliferative ability. And osteoblasts live longer than osteoclasts. Compared with osteoclasts, osteoblasts have been better potential drug target for bone loss treatment because of their biological properties. Therefore, osteoblasts were selected as the target, and the potential ability to be bone loss protectant was evaluated through the screening of proliferation activities of 9 TPENP on osteoblasts. This study differ from the medicine research and development ideas that inhibiting the differentiation or activity of osteoclast, but focus on the enhancement of osteogenesis of osteoblasts to achieve the restoration of bone homeostasis, and as a basis for the development of bone loss protectants in promote bone formation way. Through comparing the proliferation activity of 9 TPENP on osteoblasts, we found that cone scales of Korean pine has the most proliferation activity on osteoblast under the same dry weight, which showed that if the same activity is achieved, the use of the cone scales of Korean pine is less than the other 8 herbs. This result indicates that scales of Korean pine cone have had a stronger ability to alleviate bone loss compared to the herbs commonly used in *The Pharmacopoeia of the People’s Republic of China*. The result also showed that the cone scales of Korean pine have potential application value and research value.

Current studies have found that the biological activity of TPENP would increase with purity increase through isolation and purification^24,25^. In order to obtain compounds with similar chemical structure and high osteoblast proliferation activity from the total polyphenols of Korean pine, total polyphenols were isolated and purificated by macroporous resin and proliferation activity of the purified on osteoblasts was tested. Previous study has shown that macroporous resin had a good separation and purification effect on the bioactive substance in polyphenols of Korean pine^26^. In this study, the results showed that macroporous resin had good enrichment effect on polyphenols of Korean pine as a result of the column chromatography elution curve showed a sharp peak. Through the total polyphenols of Korean pine was divided into eight sections from S1 to S8, this paper discovered that S3 and S4 have higher polyphenol content while the polyphenol content in the rest of 6 sections was almost negligible, which can be concluded that compounds with osteoblast proliferation activity in total polyphenols of Korean pine are present in S3 or S4. Comparing with the EC_50_ value of the proliferation activity of S3 and S4 on osteoblasts, this paper further found that the section which had strongest proliferation activity in the total polyphenol extracts of scales from Korean pine cone was S3, and in other words, the compounds with proliferative activity of osteoblasts was mainly from S3.

In order to confirm whether S3 has the potential ability to alleviate bone loss induced by microgravity, the effect of S3 on ALP activity in osteoblasts under SMG was studied first. The results showed that the decreased ALP activity in osteoblasts induced by simulated microgravity was elevated significantly by S3 *in vitro* and present a dose-dependent manner. Then, the effects of S3 on mechanical properties of femur and cancellous in rat distal femur after HLS were studied. The results showed that the decreased mechanical properties of the femur and the changed cancellous of distal femur in rat caused by HLS were alleviated by S3 *in vivo*. In conclusion, studies *in vitro* and *in vivo* have demonstrated that S3 has the potential to alleviate bone loss under microgravity.

To investigate the mechanism of S3 in alleviating bone loss in rats under simulated microgravity, bone metabolites in rat serum were firstly tested. The results of BALP, PIPN, TRAP-5b and NTX suggest that bone loss induced by HLS was alleviated by S3 may through enhancing bone formation capacity but without inhibiting bone resorption. Secondly, in order to comfirm whether unbalanced bone homeostasis after HLS was alleviated by S3 through promoting osteogenesis of osteoblasts without inhibiting bone resorption of osteoclasts, the OPG/RANK/RANKL signaling pathway was tested in rat femur. OPG/RANK/RANKL signaling pathway is an important pathway to regulate osteoclast^27^. The results showed that S3 had no significant effect on OPG/RANKL which means S3 does not play a role in alleviating the bone loss after HLS in rats by affecting the OPG/RANK/RANKL signaling pathway. In other words, bone resorption could not be inhibited by S3 through the suppressing differentiation and activity of osteoclasts. Current study found that bone loss was caused by bone formation decreased or bone resorption enhanced^28,29^. Therefore these results inferred that bone loss was alleviated by S3 after HLS through promoting osteogenesis of osteoblasts way.

Bone loss is usually accompanied by oxidative stress^30–33^, and many experiments have confirmed that the level of oxidative stress was increased under space flight in body^34,35^. To further investigate the mechanism of S3 in alleviating bone loss in rats under simulated microgravity, the levels of oxidative stress in rat femur after HLS was tested first. The MDA results showed that increased level of oxidative stress was reduced significantly by S3. Although current study found that astronauts in space mission were subjected to oxidative damage^36^, and studies in animals also found that space flight reduced the antioxidant capacity of rat liver and promoted the development of oxidative stress^37^. In addition to microgravity, there are also strong radiations in space environment, and studies have shown that oxidative damage could cause by radiation that induced high levels of free radical in body^38,39^. This environment in space is not the same as the microgravity effects in this study. However, in addition to elevated levels of free radicals in the body, the oxidative damage may also be caused by decreased activity of antioxidant enzymes. Therefore, the activity of SOD, CAT and GSH-Px which is the key enzyme in antioxidant defense enzyme system in rat femur was detected. The results indicated that the function of the antioxidant defense enzyme system in femur decreased after HLS, which caused the increase of MDA. The expression of many enzymes in antioxidant defense enzyme system are needed the activation of the Keap1-Nrf2-ARE signaling pathway which Nrf-2 is a key regulator in body^40^. The results of Nrf-2 expression indicated that the suppression of Keap1-Nrf2-ARE signaling pathway was alleviated by S3. In this study, S3 contains polyphenolic compounds with a large number of catechin and quercetin, which molecular structure contains a large amount of covalent bonds. The larger p-π conjugated system is formed by the sp^2^ hybridization of most of these covalent bonds, and this electron-deficient center can often make the molecule have antioxidant ability. When the femur is suffered oxidative stress, the ortho phenolic group carried by catechin and quercetin is oxidized to ortho quinone structure which can alter the conformation of Keap1 by binding, thereby the expression of many downstream target genes are induced by released Nrf-2. In addition, the expression of Nrf-2 is promoted by the flavonoid structure in catechin and quercetin^41,42^. In conclusion, oxidative stress is alleviated by S3 in femur through increasing the activity of the antioxidant defense enzyme system.

Current studies have found that Wnt/β-catenin signaling pathway which played a central role in the regulation of bone remodeling could be inhibited by oxidative stress in many different cells^43–47^. To further investigate the mechanism of S3 in alleviating bone loss in rats under simulated microgravity, β-catenin, GSK3β and p-GSK3β in femur were tested first. The result indicated that the degradation of β-catenin caused by down regulation of p-GSK3β failed to enter the nucleus, resulting in the decreased expression of key osteogenesis gene was alleviated by S3 after HLS. In other words, the inhibition of Wnt/β-catenin signaling pathway was alleviated by S3 under simulated microgravity. Secondly, decreased expression of RNUX2 and OSX which can promote the differentiation and maturation of osteoblasts, and make bone homeostasis towards to osteogenesis way^48–50^, and Osteonectin, Osteocalcin, Collagen I α1 and ALP which play an important role during collagen bonding and mineralization in bone formation^51,52^ were significantly elevated by S3 after HLS. These results proved that the decreased collagen formation and mineralization of bone can be alleviated by S3 in rat under simulated microgravity.

Oxidative stress level was increased, and the transduction of Wnt/β-catenin signaling pathway was inhibited under simulated microgravity^53,54^. But the relationship between oxidative stress and transduction of Wnt/β-catenin signaling pathway in bone under microgravity has not been clarified till now. Notably, many studies on ground have shown that Wnt/β-catenin signaling pathway was inhibited by oxidative stress. But due to many signaling pathways changed under simulated microgravity, the conclusion cannot be drawn that Wnt/β-catenin signaling pathway was inhibited by oxidative stress. The results of this study showed that, first, oxidative stress induced by simulated microgravity could be alleviated by S3 in femur of rat, second the inhibition of Wnt/β-catenin signaling pathway was alleviated by S3, third, bone loss could alleviated by S3. Current research found that aging and menopause induced bone loss can be alleviated by catechin and quercetin which was contained in S3. However, reports that Wnt/β-catenin signaling pathway is activated by catechin and quercetin in bone has not been found yet. This suggests that S3 does not alleviate bone loss by simply activating the Wnt/β-catenin signaling pathway in rats under simulated microgravity. From this, the conclusion can be drawn that simulated microgravity results in oxidative stress in femur, which inhibits the transduction of the Wnt/β-catenin signaling pathway and ultimately leads to bone loss. Thus it can be seen that S3 exert a role in alleviating bone loss due to ortho phenolic group which significantly alleviate the antagonism of oxidative stress to Keap1 and flavonoid structure which significantly promote the expression of Nrf-2 that alleviate the inhibition of oxidative stress on Wnt/β-catenin signaling pathway under simulated microgravity by enhancing antioxidant enzyme system capacity through activate the Keap1-Nrf2-ARE signaling pathway.

From the presentation above, it can be concluded below. 1. S3 whom had the highest proliferation activity on osteoblasts was found through isolation and purification from the polyphenol extracts of scales of Korean pine cone which had the highest proliferation activity on osteoblasts by compared with 8 kinds of herbs which commonly used in clinic and medicinal & edible in *The Pharmacopoeia of the People’s Republic of China*. 2. S3 has the potential to alleviate the bone loss in rats induced by simulated microgravity through *in vitro* and *in vivo* studies. 3. Simulated microgravity induced bone loss by causing oxidative stress which inhibited Wnt/β-catenin signaling pathway in rat. 4. Bone loss can be alleviated by S3 with structure of catechin, quercetin and the ortho phenolic group through the activation of Wnt/β-catenin signaling pathway which inhibited by oxidative stress under simulated microgravity. At last, the potential weightlessness bone loss protectant S3 have found in the polyphenol extracts of scales of Korean pine cone through the screening of proliferation activity of TPENP on osteoblasts. The medicinal value of cone scales of Korean pine has been developed, and a new approach for developing the protectants for bone loss in microgravity has been provided in this study.

## Materials and methods

### Reagent and natural products

The fetal bovine serum and Medium (α-MEM) were purchased from HYCLONE. Pentobarbital sodium, Na_2_CO_3,_ Folin-Ciocalteu reagent, Thiazole blue (MTT), Dimethyl sulfoxide (DMSO) were purchased from Sigma. The 9 natural products used in this study included morinda, drynaria, rehmannia, epimedium, eucommia, astragalus, kudzu, psoralen and cone scales of Korean pine. All the herbal medicines were bought from Harbin Tongrentang Traditional Chinese Medicine Pharmacy, and cones scales of Korean pine were from Daxinganling of China.

### Culture of primary osteoblast

3-day new born SD rat were sacrificed to obtain osteoblasts for culture. Calvarias were excised, and all attached tissues were carefully removed under. After that, the calvarias were cut into 0.5 mm^2^ pieces and incubated with Trypsin Solution (Solarbio, China) at 37 °C for 15 min. Then the calvarias were incubated with collagenase I (0.1%PBS solution)(Solarbio, China) at 37 °C after washed with PBS 3 times. One hour later, the calvarias were put into flask with α-MEM (10% serum and 1% mycillin) after washed with PBS 3 times. The calvarias were removed 5 days later after the flask incubate at 37 °C.

### Determination of polyphenols concentration

The content of polyphenol was tested by the improved Folin-Ciocalteu method. Folin-Ciocalteu reagent (1 M, 0.5 mL) was added into the the sample solutions (0.5 mL). After mix for 5 minutes, Na_2_CO_3_ (1.0 mL, 10% (w/v)) and distilled water (1 ml) was added. The mixtures were incubated at room temperature without light for 2 hours. Distilled water was used for control instead of the sample solution. Spectrophotometer (analytikjena, Germany) was used to measure the absorption value (760 nm) of mixtures. Gallic acid (0–70 μg/mL) was used as the standard solution to make the standard curve (see Supplementary Fig. S2). The regression equation was y = 0.01222x + 0.012256 (R^2^ = 0.9991, n = 6). In this equation, y is the absorption value of gallic acid and x is the concentration (μg/mL). Concentration of polyphenol could be figured out by the regression equation and the dilution multiple of sample solution.

### Total polyphenol extracts of 9 natural products

The 9 natural products were crushed into powders. Then ethanol solution (ethanol:water = 5:3) was mixed with the powders (1:20, W/V), placed at 4 °C. After 5 h, the mixtures were placed under ultrasound (40 °C, 500 W) for 30 min. Three times later, supernatants were collected and dried into powders by vacuum freeze dryer (NINGBO SJIALAB EQUIPMENT CO., LTH, China). The dry powders dissolved with distilled water (1:100, W/V). Then petroleum was added to the solutions (1:1, V/V). After Stir for 2 hours and stand for 1 hour, the aqueous phase was separated and mixed with Chloroform and n-butanol (aqueous phase: Chloroform: n-butanol = 6:5:1, V/V) for 2 hours. Then, supernatants were collected and dried into powders by vacuum freeze dryer.

### Comparison of osteoblats proliferation activity of 9 TPENP

The osteoblasts were put into 96-well plate (5000 cell/well). After 1 day incubation (37 °C), the medium was replaced with α-MEM with TPENP in different concentration (0.1, 0.2, 0.3, 0.4, 0.5, 0.6, 0.7, 0.8, 0.9, 1.0 mg/mL) and then incubated at 37 °C for 2 days. Then medium (contained 10 mg/mL MTT, 200 μL) was put into the each well and incubated at 37 °C for 4 h after washed with PBS 3 times. The medium was replaced with DMSO (200 μL). The absorbance (490 nm) of each well was measured after vibrated for 10 min at room temperature in microplate reader. The proliferation rate is calculated as follow formula. Proliferation rate = (sample absorbance-control absorbance)/control absorbance × 100%.

The EC_50_ values of proliferation activity of 9 TPENP on osteoblats were obtained through Calcusyn software (version 2.0, Biosoft, USA).

### The purification of total polyphenol extracts from cones scales of Korean pine

The powder of total polyphenol extracts from cone scales of Korean pine was dissolved into distilled water (1 mg Gallic acid equivalent/ml). Macroporous resin (D101) was used to isolation and purification of polyphenols. Ratio between diameter of chromatographic column and height was 1: 30. The sample volume is 1/3 column volume. After the sample was added, 2 column volume was eluted by distilled water, and then, 2 column was eluted by ethanol solution (ethanol:water = 5:3). And eluent was collected every 1/4 of the column volume. At the end, a total of 8 eluents (S1–S8) were collected. The results of the identification of the compounds in S3 are shown in Supplementary Fig. S3, including catechin-3-O-glucose, Catechin-3-o-mannoside, Roseoside I, dihydroquercetin, Methyl quercetin rhamnoside, (2E, 4E)-5-{6-(β-D-Glucopyranosyloxy) methyl-1-hydroxyl-2, 6-dimethyl-4-oxo-2-cyclohexen-1-yl}-3- methyl-2, 4-pentadienoic acid.

### Simulated microgravity effect

The simulated microgravity effect of osteoblasts was based on the method of Wei, L., *et al*.^55^. The third passage of primary osteoblasts was inoculated in the six-well plate where blood cover film was covered. After 1 day incubation (37 °C), blood cover films were placed in 2D-RWVS (rotation speed at 30r/min) and cultivated according to CK (medium without S3), SMG (medium without S3), SMG + S3(L) (medium with S3, 2.23 μg/mL), SMG + S3(M) (medium with S3, 4.46 μg/mL and SMG + S3(H) (medium with S3, 6.69 μg/mL). After 72 hours, osteoblasts were taken out and used for the ALP test.

Male SD rats (6 weeks, 200 g) were obtained. All animal experiments were performed in accordance with the Care and Use of Laboratory Animals of Chinese society of experimental animal. The protocol is approved by the ethical committee of the experimental animal welfare of China Astronauts Research and Training Center. The 50 rats were divided into five groups (10 rats for each group): CK, HLS, HLS + S3(L), HLS + S3(M) and HLS + S3(H). HLS was performed in each group except CK. CK and HLS were gavaged with distilled water, HLS + S3(L), HLS + S3(M) and HLS + S3(H) were gavaged with S3 for the dose of 15 mg/kg/d, 37.5 mg/kg/d and 60 mg/kg/d, respectively, since the first day that HLS was performed. 30 days later, blood sample was obtained after rats were narcotized by pentobarbital sodium (30 mg/kg) intraperitoneal injection. Rat bilateral femurs were harvested after the rats were sacrificed. The left femur was fixed with ethanol (ethanol:water = 7:3) after muscle and cartilage tissue had been removed. The mechanical property of left femur was tested after Micro-CT had been scanned. The both ends of right femur were cut out, and then the bone marrow of right femur was washed away by PBS solution. After that, the right femur was crushed into powder under liquid nitrogen. Total protein which was used for biochemical analysis and western blot test was extracted from the powder using RIPA buffer (Solarbio, China) (4:5, w/v). Total RNA which was used for qRT-PCR test was extracted from the powder using Trizol (ambion, USA) (4:5, w/v).

### ALP activity in osteoblasts

Osteoblasts were digested with Trypsin Solution from Blood cover films. ALP activities in osteoblasts were determined according to the instruction of ALP assay kit (Nanjing Jiancheng Bioengineering Institute, China) and BCA protein assay kit (Nanjing Jiancheng Bioengineering Institute, China). The ALP activities are calculated as follow formula. ALP activity = ALP activity of sample/total protein of sample.

### Mechanical property of rat femur

The mechanical property of left femur of rat was measured by 3-point bending test. A universal testing machine (Brookfield, USA) was used to support the platform. The span between 2 load points was 15 mm. The femurs were put on the support platform in the same direction with a load (0.05 mm/s) on the center point until fracture. The mechanical properties of the femurs were determined through bending curves which was obtained by the femoral load at the same time, including elastic modulus, stiffness, maximum strength, energy.

### Micro-CT

Microstructural analysis of cancellous bone in the distal femur was performed by micro-CT (Scanco Medical, Switzerland). The femur was vertically aligned with the scanning axis. The spatial resolution of the sample scan was set to 5 μm per voxel. The femur was identified with semi-automatically drawn contour at each two dimensional (2-D) section. The region of interest (ROI) was distal femoral growth plate above 2.1 cm which determined using 200 serial sections. 3D reconstructed images of ROI were obtained and analyzed through image analysis program of the µ-CT workstation.

### Biochemical analysis

The serum concentrations of BALP, PINP, TRAP-5b, NTX and the concentration of OPG, RANKL in femur, were determined using an enzyme-linked immunoassay detection kit (SHANGHAI SUER BIOLOGICAL TECHNOLOGY CO.,LTD, China). The value of OPG/RANKL is calculated as follow formula. OPG/RANKL = (concentration of OPG in sample/concentration of total protein in sample)/(concentration of RANKL in sample/concentration of total protein in sample). MDA level, activity of SOD, CAT, GSH-Px was determined using a Malondialdehyde (MDA) assay kit, Superoxide Dismutase (SOD) assay kit, Catalase (CAT) assay kit, Glutathione Peroxidase (GSH-PX) assay kit, respectively. The kits were all purchased from Nanjing Jiancheng Bioengineering Institute, China. The MDA level and the activity of SOD, CAT, GSH-Px are calculated as follow formula. MDA and activity of SOD, CAT, GSH-Px = MDA level, activity of SOD CAT and GSH-Px in sample, respectively/total protein in sample.

### Western Blot

The protein sample (40 μg) was taken for constant-voltage electrophoresis (120 V). Protein belt was transferred to the 0.22 μM PVDF membranes when the minimum molecular weight band of marker reached lower edge of 12% SDS-PAGE. Then the membranes were immersed into TBST solution (Tris-buffered saline containing 0.1% Tween-20(v/v), 5% milk powder (w/v)) for 2 h. After washed with TBST (Tris-buffered saline containing 0.1% Tween-20(v/v)) 3 times, the membranes were put into TBST solution with primary antibodies (0.02%, v/v) and kept in 4 °C for one night. Primary antibodies including β-catenin (ab16051), GSK3-β (ab131356), P-GSK3-β (ab131097), β-actin (ab8227) (All primary antibodies were obtained from Abcam, USA). After washed with TBST three times, the membranes were incubated with the secondary anti-rabbit antibodies (ZSGB-BIO, China) at the concentration of 1:5000 for 90 min at room temperature. The membranes were washed with TBST 3 times. Then the protein band was obtained by X-ray film exposure method after ECL (Solarbio, China) was dropped on the membranes in a dark room. Quantity One software (version 4.6.2, Bio-Rad, USA) was used to analysis the bands.

### Quantitative real-time PCR

Quantitative real-time PCR was performed to detect the expression levels of several genes (i.e., NRF-2, RUNX2, OSX, Osteonectin, Osteocalcin, ALP, collagen Iα1). The reverse transcription reaction was performed using 2 μg of total RNA that was reverse transcribed into cDNA using PrimeScript TM RT reagent Kit (TAKARA BIO INC, Japan), quantitative real-time PCR analysis was carried out using an ViiA 7 system (Applied Biosystems, USA). Quantitative real-time PCR analysis assays were carried out in triplicate on FastStart Universal SYBR Green Master (Roche, USA) following the commended protocols. All amplifcations were normalized against GAPDH. The data were analyzed via the relative Ct (ΔΔCt) method. The primer sequences were designed by Comate Bioscience Co., Ltd., Jilin, China.

### Statistical analysis

All data were statistically analyzed by SPSS 17.0 software. The result was showed as the means ± SD of at least 3 independent experiments. One-way ANOVA was used to compare differences among groups and *P* values < 0.05 were considered statistically significant.

## Electronic supplementary material

Supplementary information

## Data Availability

The datasets generated during and/or analyzed during the current study are available from the corresponding author on reasonable request. All data generated or analyzed during this study are included in this published article (and its Supplementary Information files).
