# Supplementary material for: Polyphenols (S3) Isolated from Cone Scales of Pinus koraiensis Alleviate Decreased Bone Formation in Rat under Simulated Microgravity
Source: Sci Rep. 2018 Aug 24;8:12719. doi: 10.1038/s41598-018-30992-8 (PMC6109125; doi:10.1038/s41598-018-30992-8)
Supplement: Supplementary file 1 — Supplementary information [file 41598_2018_30992_MOESM1_ESM.pdf]

Polyphenols (S3) Isolated from Cone Scales of Pinus koraiensis Alleviate Decreased Bone Formation in Rat under Simulated Microgravity

Yan Diao, Bin Chen, Lijun Wei & Zhenyu Wang

Supplementary Fig. S1

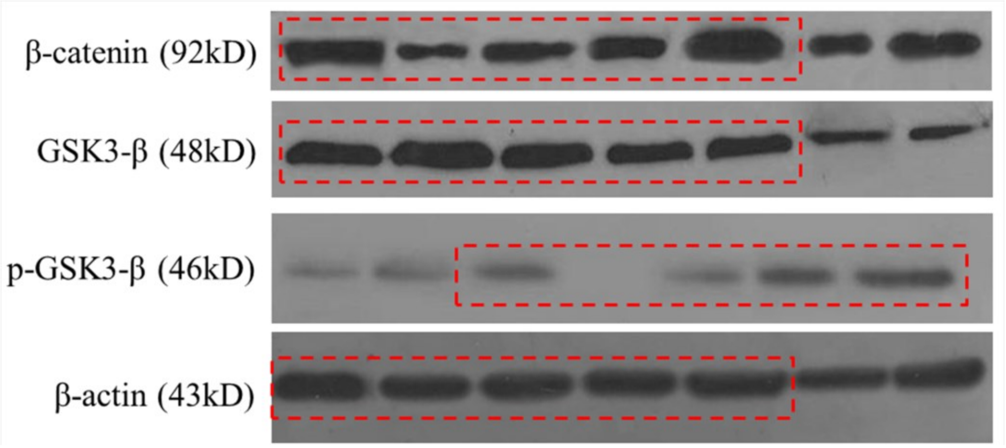

Supplementary Fig. S1 Uncropped, unprocessed images of blots and gels.

Supplementary Fig. S2

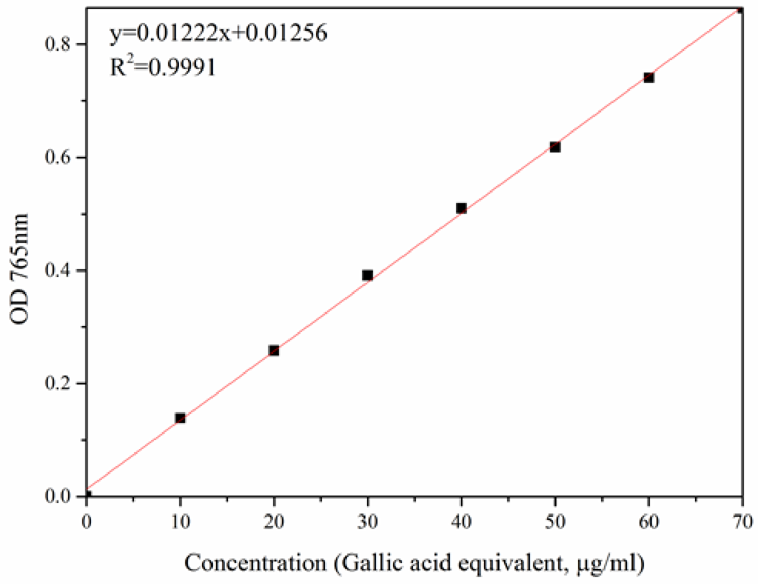

Supplementary Fig. S2 The standards curve of polyphenol (Gallic acid equivalent).

Supplementary Fig. S3

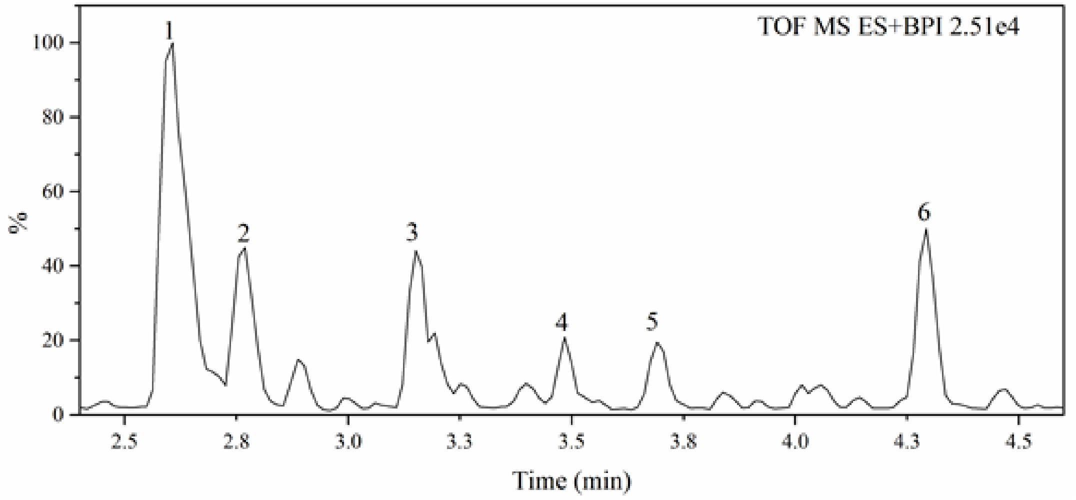

Supplementary Fig. S3 Chromatogram of polyphenols from cones scales of pinus koraiensis obtained by UPLC-ESI-MS in positive ion mode. 1: Catechin-3-O-glucose, 2: Catechin-3-o-mannoside, 3: Roseoside I, 4: Dihydroquercetin, 5: Methyl quercetin rhamnoside, 6: (2E,4E)-5-{6-[(β-D-Glucopyranosyloxy)methyl]-1-hydroxy-2,6-dimethyl-4-oxo-2-cyclohexen-1-yl}-3-methyl-2,4-pentadienoic acid

Supplementary Fig. S4

The expression of OPG and RANKL in femur of rat was tested (Fig. S4). The OPG/RANKL value decreased significantly in femur of rat after HLS, and there were no significant change of all three dose of S3 on the OPG/RANKL value.

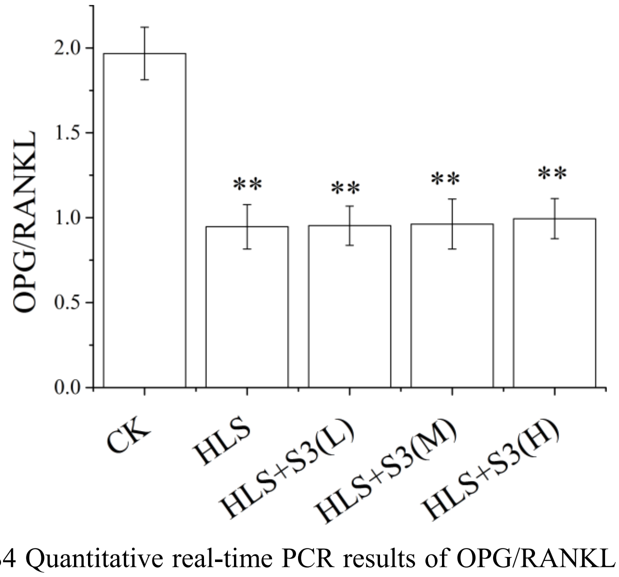

Supplementary Fig. S4 Quantitative real-time PCR results of OPG/RANKL in femur of rats. CK: Ground control, SMG: simulated microgravity, S3(L): low dose of S3, S3(M): middle dose of S3, S3(M): high dose of S3. The statistical results shown represent the means±SD; (n = 3). vs. the HLS group # P<0.05, ## P<0.01; vs. the CK group * P<0.05, ** P<0.01.

Supplementary Fig. S5

The results shown that under simulated microgravity for 72 h, ALP activity in osteoblasts was significantly decreased. S4 could elevate ALP activity at a way of dose-effect, however, high-doses S4 still failed to achieve ALP activity to normal levels in osteoblast under simulated

microgravity.

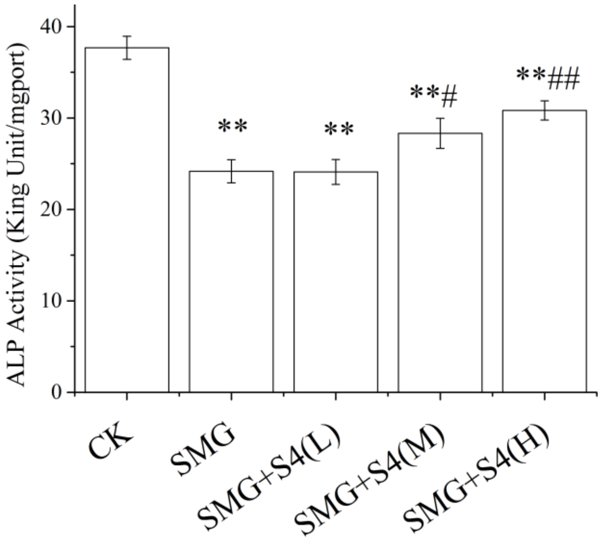

Supplementary Fig. S5 Decreased ALP activity was alleviated by S4 in osteoblasts under simulated microgravity. 2 direction rotating (2D-RWVS), China Astronauts Research and Training Center) was used to set up a simulated microgravity model in vitro. CK: Ground control, SMG: simulated microgravity, S4(L): low dose of S4, S4(M): middle dose of S4, S4(M): high dose of S4. The statistical results shown represent the means±SD; (n = 3). vs. the SMG group # $P<0.05$, ## $P<0.01$; vs. the CK group * $P<0.05$, ** $P<0.01$.
